# Elective Transjugular Intrahepatic Portosystemic Shunt Using Viatorr Stent-Grafts: A Single-Center Experience from China

**DOI:** 10.5334/jbsr.2741

**Published:** 2022-06-29

**Authors:** Yu-Hua Li, Yue-Meng Wan, Hua-Mei Wu, Song-Quan Huang

**Affiliations:** 1 Kunming medical university Second Hospital, CN

**Keywords:** transjugular intrahepatic portosystemic shunt, Viatorr TIPS stent-grafts, shunt dysfunction, variceal bleeding, hepatic encephalopathy

## Abstract

**Background and Aims:**

Transjugular intrahepatic portosystemic shunt (TIPS) is a well-established approach for the management of variceal bleeding, refractory ascites, hepatic hydrothorax, and preoperative treatment of portal hypertension prior to major abdominal surgery in patients with compensated cirrhosis, and so on. This study aimed to investigate the safety and long-term efficacy of TIPS implantation using Viatorr TIPS stent-grafts.

**Material and Methods:**

A cohort of 59 patients undergoing TIPS placement using Viatorr TIPS stent-grafts were included, and the periprocedural events, and long-term mortality, shunt dysfunction, variceal rebleeding and incidence of hepatic encephalopathy (HE) were analyzed.

**Results:**

The technical success rate was 100%. The median portosystemic pressure gradient was reduced from 21 mmHg (interquatile range: 19–25) to 13 mmHg (interquatile range: 10–16) before and after TIPS, leading to a hemodynamic success rate of 72.9%. The cumulative rate of overall mortality was 34.2% at five years, and direct bilirubin (hazard ratio [HR] = 1.336, 95% confidence interval [CI]: 1.050–1.700, P = 0.018) and post-TIPS right atrial pressure (HR = 1.238, 95% CI: 1.015–1.510, P = 0.035) were independent predictors for mortality. The cumulative rates of shunt dysfunction and variceal rebleeding were 11.0% and 28.3% at five years, respectively, and portal venous pressure gradient (HR = 2.572, 95% CI: 1.094–6.047, P = 0.030) was the only independent predictor for shunt dysfunction. The cumulative four-year HE-free rate was 48.6%. No severe adverse event was noted during TIPS procedures.

**Conclusion:**

Elective TIPS implantation using Viatorr TIPS stent-grafts is generally safe, and the long-term efficacy is favorable for the treatment of cirrhotic patients with recurrent variceal bleeding or refractory ascites.

## Introduction

Liver cirrhosis may lead to portal hypertension (PH)-related complications, including variceal bleeding from esophageal and/or gastric varices, and refractory ascites (RA), which represent the major causes of hospital admission. Transjugular intrahepatic portosystemic shunt (TIPS) can reduce portal hypertension, and is thus a well-established therapy for recurrent variceal bleeding and RA in cirrhotic patients [1].

Since its first application in dogs by Rösch et al. [2] reported in 1971 and the first insertion in human in 1989 [3], the TIPS technique has gradually evolved from using bare metal stents initially to using expanded polytetrafluoroethylene (ePTFE)-covered stent-grafts nowadays, and become a generally accepted treatment for PH-related complications [4,5]. Along with the development of material science, ePTFE-covered stent-grafts also transitioned from generic ones (Fluency Plus, Bard Peripheral Vascular) [6] to the Viatorr TIPS Endoprosthesis (W. L. Gore & Associates) [7,8], posing great influence on the role of TIPS implantation for the management of PH-related complications. In China, the Viatorr TIPS Endoprosthesis was not available until October 2015, about a decade later than western countries [9], at which time TIPS was mainly created using generic Fluency Plus stent-grafts with or without bare metal stents. Despite there are numerous reports about the efficacy and safety of TIPS implantation using Viatorr TIPS stent-grafts from Western countries, scarce information is available in the literature about the use of Viatorr TIPS stent-grafts in Chinese patients. This is invaluable since Chinese patients differed greatly from their counterparts in western countries in terms of etiology of liver disease, huge volume of population, antropometric data, and so on [10,11].

Therefore, we performed this retrospective and single-centre study to evaluate the safety and long-term efficacy of elective TIPS implantation using Viatorr TIPS stent-grafts for treatment of cirrhotic patients with recurrent variceal bleeding or RA.

## Materials and Methods

The study approval was granted by ethics committee of the Second Affiliated Hospital of Kunming Medical University. Informed consent for medical research was waived because the patients’ data were collected retrospectively, and anonymised before analysis.

### Study Population

Between August 2016 and October 2021, 72 consecutive patients with liver cirrhosis and recurrent variceal bleeding (n = 71) and RA (n = 1) who underwent TIPS implantation were screened from our electronic database. Exclusion criteria were follow-up loss (n = 5), TIPS created within 30 days (n = 4) or outside of our hospital (n = 2) and refusal to participate (n = 2). In total, only 59 patients were included in this study.

### Tips Procedures

**Figure 1:**
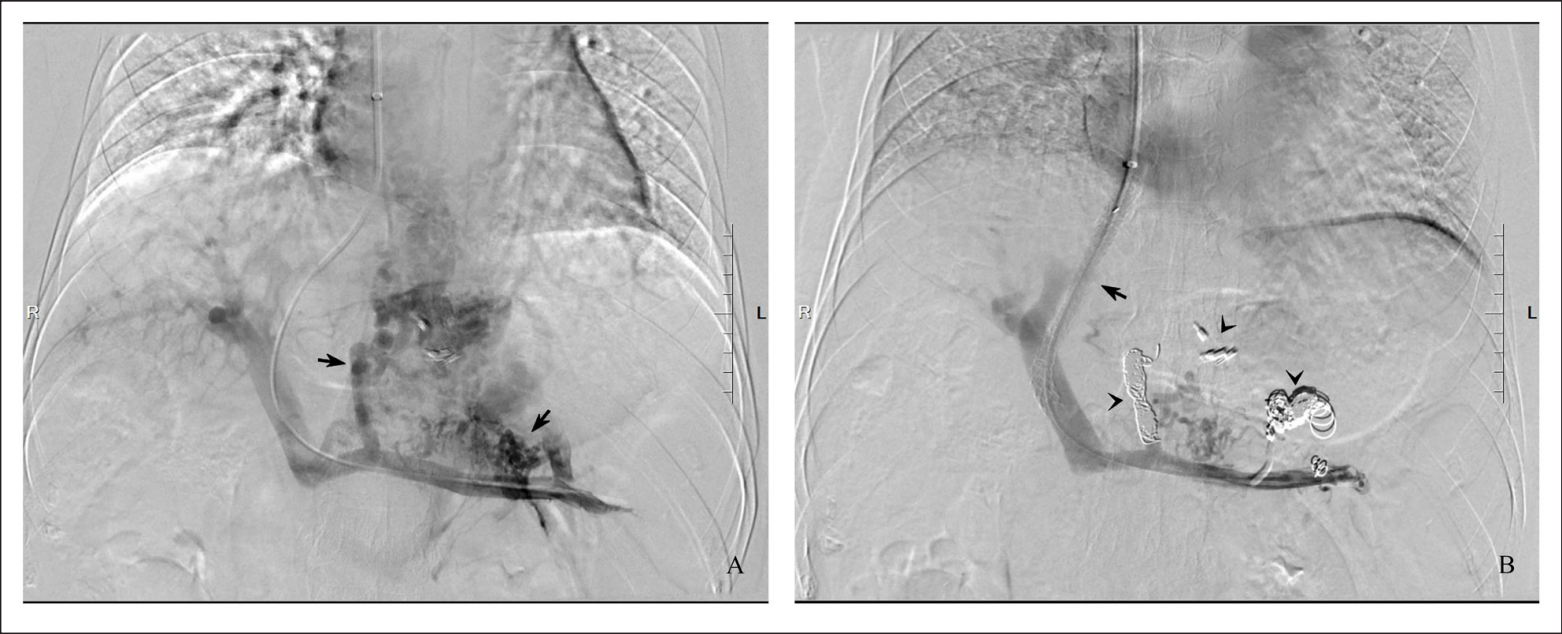
Venogram demonstrating a 56-year-old male patient with dilated collaterals (**A**, arrow) undergoing implantation of an 8 mm × 20/70 mm Viatorr TIPS stent-graft (B, arrow) and collateral embolization using spring coils (**B**, arrowhead).

TIPS insertion was conducted as previously described in detail [12,13]. At the discretion of operating interventional radiologists, all TIPS procedures were conducted using ePTFE-covered legacy Viatorr TIPS stent-grafts (VTS; Viatorr®, W.L. Gore & Associates, Inc. Flagstaff, Arizona, USA; Figure 1A) with a specification of 8 mm in nominal diameter and 50–70 mm/20 mm in length (such as 8 mm × 50–70 mm/20 mm). An additional bare metal stents (Angiomed subsidiary of C.R. Bard, New Jersey, USA) was inserted coaxially when the initial Viatorr TIPS stent-graft fell short to cover the entire punctured hepatic parenchyma. Dilated collaterals, including gastric coronary vein and/or short gastric vein (Figure 1B), were embolized using spring coils of different sizes (Cook Inc., Bloomington, IN, USA; Figure 1C). TIPS patency was checked at three, six months and then at 6–12 monthly intervals using Doppler ultrasonography or computerized tomography portal venography (CTPV), but it was confirmed by direct portal venography. The choice between Doppler ultrasonography and CTPV was up to the patients’ managing physicians, while the invasive procedure of direct portal venography was only performed when a patient had both clinical recurrence of portal hypertension-related complications (such as variceal rebleeding, deterioration of ascites) and abnormal ultrasonography (for an example, slowed or undetectable blood flow within the TIPS) in combination with abnormal CTPV (lack of contrast filling within the shunt). If TIPS dysfunction was confirmed, balloon angioplasty was performed or an extra ePTFE-covered stent-graft or bare metal stent was implanted.

### Treatment, Follow-Up and Data Collection

All patients were treated as previously described before and after TIPS placement [12,13]. Follow-up visits were conducted through telephone calls or Wechat, outpatient and/or hospital visits that were scheduled at three, six months and then at 6–12 monthly intervals or any time when they felt unwell. At hospital visits, patients were subjected to clinical examination, blood testing and TIPS patency checkup as described previously. Information on the clinical outcomes, including shunt dysfunction, variceal rebleeding, HE, liver transplantation and mortality, were recorded. Data were collected prospectively, including patients’ demographic characteristics, clinical outcomes, laboratory findings and technical information of TIPS procedures and any re-interventions, and adverse events. Model for end-stage liver disease (MELD) and Child-Turcotte-Pugh (CTP) scores were calculated at baseline to evaluate the severity of liver disease [14]. Follow-up was continued until the last clinical assistance or recurrence of symptoms of PH, liver transplantation, death or study end in November 2021.

### End Points

Primary end point was to determine the overall mortality and its predictors. Secondary end-points were to determine the rates of technical and hemodynamic success, shunt dysfunction, variceal rebleeding, post-TIPS HE and safety profile.

### Definitions

Refractory ascites referred to persistent ascites despite therapeutic paracentesis, appropriate dietary salt restriction and maximum tolerable treatment with diuretics [15]. Elective TIPS implantation was defined as stent implanted after 72 hours of variceal bleeding [16] that was initially controlled by endoscopy. Technical success was defined as the creation of a patent TIPS between the hepatic vein and branch of the portal vein in the presence of patent portal and hepatic vein, while hemodynamic success referred to the reduction of the portosystemic pressure gradient (PSPG) to 12 mmHg or less, or a reduction of at least 20% from the initial value [17]. TIPS dysfunction referred to stenosis ≥50% or occlusion of the TIPS [18]. Variceal bleeding was defined according to Baveno VI criteria [19]. The occurrences of HE, such as lethargy, apathy, disorientation, inappropriate behavior, somnolence and obvious personality changes, were documented in detail, and after repeated confirmation, the stage and degree of HE were assessed according to the West Haven Criteria [20].

### Statistical Analyses

Statistical analysis was performed using SPSS version 22 (SPSS Inc., Chicago IL). Quantitative data were expressed as median and interquartile range (Q1–Q3), and categorical variables were presented as numbers and percentage. Cumulative incidences of the major events following TIPS, namely shunt dysfunction, variceal rebleeding, HE and mortality, were calculated and plotted with the Kaplan-Meier curve from the date of TIPS insertion to the date of censoring (follow-up loss, liver transplant, death or study closure, whichever came first). Univariate Cox proportional hazard regression was performed to analyze prognostic predictors of each post-TIPS major event. Multivariate Cox proportional hazard regression analysis was subsequently performed to measure the independent contribution of each factor on these events. Two-sided P values were calculated and statistical significance was defined as P < 0.05.

## Results

### Patient Characteristics at Baseline

Detailed characteristics and laboratory results of all 59 patients at baseline were shown in Table 1. The median age was 52 (47–59) years with the majority being male (61.0%). Etiology of liver cirrhosis was mainly hepatitis B and C virus infection, accounting for 42.4% and 16.9%, respectively. The indication for TIPS creation was recurrent variceal bleeding (98.3%) and RA (1.7%). Two (3.4%) patients with Budd-Chiari syndrome (BCS) received implantation of a covered VTS in combination with a bare metal stent. TIPS was created from middle hepatic vein to left portal branch in 45 (76.3%) patients, or 45 (76.3%) patients had left portal branch puncture, as determined by both the patients’ anotomic conditions and the operators’ habit and technique. Twenty-eight (47.5%) patients presented with Child class B, and the median CTP score was 8 (6–9) and median MELD score was 9.33 (6.82–11.52).

**Table 1:** Baseline characteristics of the study population.


VARIABLES	ALL PATIENTS (N = 59)

Age (yr)^a^	52(47–59)

Gender: male, n(%)	36(61.0%)

Etiology, n(%)	

Cryptogenic	4(6.8%)

Alcoholic	9(15.3%)

Hepatitis B virus	25(42.4%)

Hepatitis C virus	10(16.9%)

Autoimmune liver disease	9(15.3%)

Budd-Chiari syndrome	2(3.4%)

Comorbidity, n(%)	

Diabetes mellitus	13(22.0%)

Hypertension	1(1.7%)

Hepatocellular carcinoma	0(0.0%)

Baseline portal vein thrombosis, n(%)	19(32.2%)

Ascites, n(%)	

None	15(25.4%)

Mild	20(33.9%)

Moderate to severe	24(40.7%)

Child class, n(%)	

A	19(32.2%)

B	28(47.5%)

C	12(20.3%)

TIPS indication, n(%)	

Esophageal variceal bleeding	14(23.7%)

Gastroesophageal variceal bleeding	44(74.6%)

Refractory ascites	1(1.7%)

Portal branch puncture, n(%)	

Left branch	45(76.3%)

Other portal vein	14(23.7%)

Bare stent use, n(%)	2(3.4%)

Stent connection, n(%)	

From middle hepatic vein to left portal branch	45(76.3%)

From middle hepatic vein to right portal branch	1(1.7%)

From right hepatic vein to right portal branch	11(18.6%)

From IVC to portal vein	2(3.4%)

Pre-TIPS portal venous pressure (mmHg)^a^	33(28–36)

Post-TIPS portal venous pressure (mmHg)^a^	23(21–26)

Post-TIPS right atrial pressure (mmHg)^a^	11(7–13)

Portal venous pressure gradient (mmHg)^a^	9(6–11)

White blood cell count (3.5–9.5 × 109/L)^a^	2.87(2.05–3.88)

Hemoglobin (female:115–150 g/L; male:130-175 g/L)^a^	84(74–100)

Platelet count (125–350 ×109/L)^a^	60(47–79)

Prothrombin time (10.0-16.0 s)^a^	16(14.9–17.6)

International normalized ratio^a^	1.32(1.19–1.48)

APTT (28.0-43.5 s)	39.2(36.4–46.3)

Albumin (35-50 g/L)^a^	30.6(27.2–36.4)

Alanine aminotransferase (5-40 U/L)^a^	24(17–29)

Aspartate aminotransferase (8-40 U/L)^a^	31(23–46)

Cholinesterase (5000-12000 U/L)^a^	3400(2478–4264)

Total bile acid (0.0-10.0 µmol/L)^a^	13.8(7.8–20.4)

Total bilirubin (3.4-20.5 µmol/L)^a^	25.4(18.0–34.8)

Direct bilirubin (0.0-6.8 µmol/L)^a^	12.0(7.0–18.4)

Total cholesterol (3.49-5.18 mmol/L)^a^	2.64(2.18–3.36)

Triglyceride (0.25-1.71 mmol/L)^a^	0.75(0.64–1.10)

Creatinine (53-97 µmol/L)^a^	68(55–79)

Child-turcotte-pugh score ^a^	8(6–9)

Model for end stage liver disease score ^a^	9.33(6.82–11.52)

Follow-up duration (month) ^a^	38(29–45)


^a^ Median (interquatile range); IVC, inferior vena cava; TIPS, transjugular intrahepatic portosystemic shunt; APTT, activated partial thromboplastin time.

### Technical Outcome

TIPS creation was technically successful in all 59 patients (100%). The median PSPG before and after TIPS was 21 mmHg (19–25) and 13 mmHg (10–16), respectively. As a result, hemodynamic success defined as a PSPG reduction at least 20% from the initial value was only attained in 43 patients (72.9%).

### Mortality and Predictors

**Figure 2:**
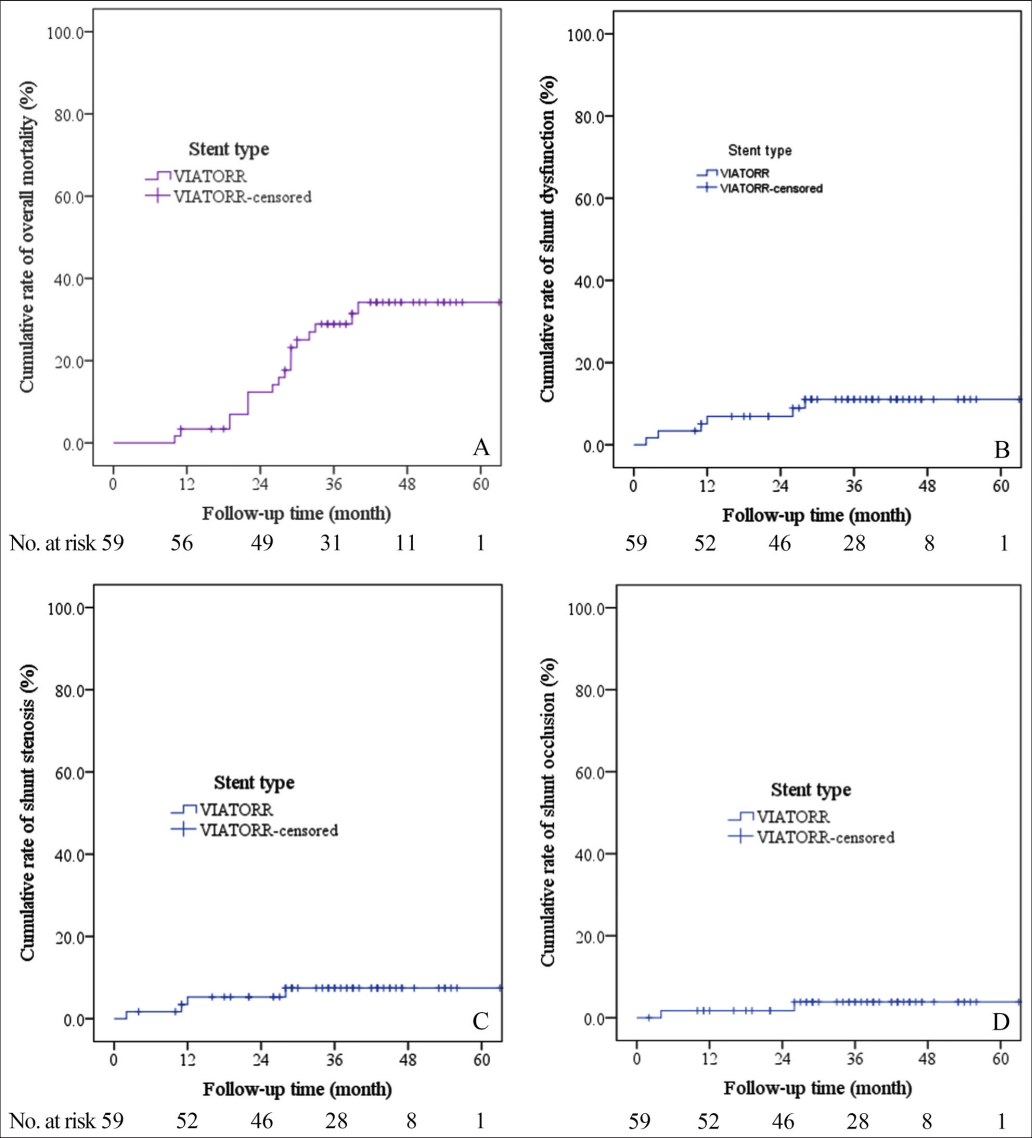
Cumulative rates of overall mortality **(A)**, shunt dysfunction **(B)**, shunt stenosis **(C)**, and shunt occlusion **(D)**.

During a median follow-up period of 38 months (IQR 29–45 months), 18 patients (30.5%) died due to the following causes: liver failure (n = 8), sepsis (n = 4), severe HE (n = 2), recurrent bleeding (n = 2), hepatocellular carcinoma (n = 1) and cervical cancer (n = 1). And two (3.4%) patients underwent liver transplantation at three and five months after TIPS, respectively. Thus, the cumulative rate of overall mortality was 3.4%, 12.3%, 28.9%, 34.2% and 34.2% at 1, 2, 3, 4 and 5 years, respectively (Figure 2A). In univariate analysis, pre-TIPS portal venous pressure, post-TIPS right atrial pressure, hemoglobin, total and direct bilirubin, international normalized ratio (INR), prothrombin time (PT), CTP score, Child class, and bare stent use were significantly associated with mortality. However, only direct bilirubin (hazard ratio [HR] = 1.336, 95% confidence interval [CI]: 1.050–1.700, P = 0.018) and post-TIPS right atrial pressure (HR = 1.238, 95% CI: 1.015–1.510, P = 0.035) remained significantly associated with mortality in multivariate analysis (Table 2).

**Table 2:** Univariate and multivariate analyses of predictors for mortality.


BASELINE VARIABLES	UNIVARIABLE ANALYSIS			MULTIVARIABLE ANALYSIS		
	
	HR	95% CI	P VALUE	HR	95% CI	P VALUE

Direct bilirubin (µmol/L)	1.079	1.039–1.121	0.000	1.336	1.050–1.700	0.018

Post-TIPS right atrial pressure (mmHg)	1.151	1.007–1.351	0.039	1.238	1.015–1.510	0.035

INR	11.819	2.815–49.627	0.001			

Child class^#^	2.305	1.234–4.307	0.009			

Child score	1.382	1.049–1.822	0.022			

PT (s)	1.337	1.109–1.613	0.002			

Pre-TIPS portal venous pressure (mmHg)	1.095	1.012–1.186	0.025			

Total bilirubin (µmol/L)	1.035	1.012–1.059	0.003			

Hemoglobin (g/L)	0.968	0.943–0.993	0.013			

Bare stent use*	0.167	0.038–0.746	0.019			


HR, Hazard ratio; CI, confidence interval; TIPS, transjugular intrahepatic portosystemic shunt; PT, prothrombin time; INR, international normalized ratio; Child class ^#^: 1 = A, 2 = B, 3 = C; Bare stent use*: no bare stent use *vs*. bare stent use.

### Shunt Dysfunction and Predictors

In total, two patients (3.4%) developed TIPS occlusion, and four (6.8%) developed shunt stenosis, rendering a cumulative shunt dysfunction rate of 6.9%, 6.9%, 11.0%, 11.0% and 11.0% at 1, 2, 3, 4 and 5 years (Figure 2B), respectively. Meanwhile, the cumulative shunt stenosis rate was 5.3%, 5.3%, 7.5%, 7.5% and 7.5% (Figure 2C), and the cumulative TIPS occlusion rate was 1.7%, 1.7%, 3.9%, 3.9% and 3.9% (Figure 2D) at 1, 2, 3, 4 and 5 years, respectively. Notably, the two cases of TIPS occlusion occurred at the proximal part (hepatic end) of Viatorr stent-grafts, while two cases of shunt stenosis occurred in the proximal part (hepatic end), one case in the middle part, and the remaining one case in the distal part (portal end). The etiology of shunt occlusion was stent kinking and thrombosis at hepatic end (n = 2), and shunt stenosis was caused by stent kinking and thrombosis at hepatic end (n = 2), middle portion (n = 1) and portal end (n = 2). In univariate analysis, portal venous pressure gradient, stent connection and etiology were markedly associated with shunt dysfunction. In multivariate analysis, only portal venous pressure gradient (HR = 2.572, 95% CI: 1.094–6.047, P = 0.030) remained an independent predictor for shunt dysfunction (Supplementary Table 1).

### Variceal Rebleeding and Predictors

**Figure 3:**
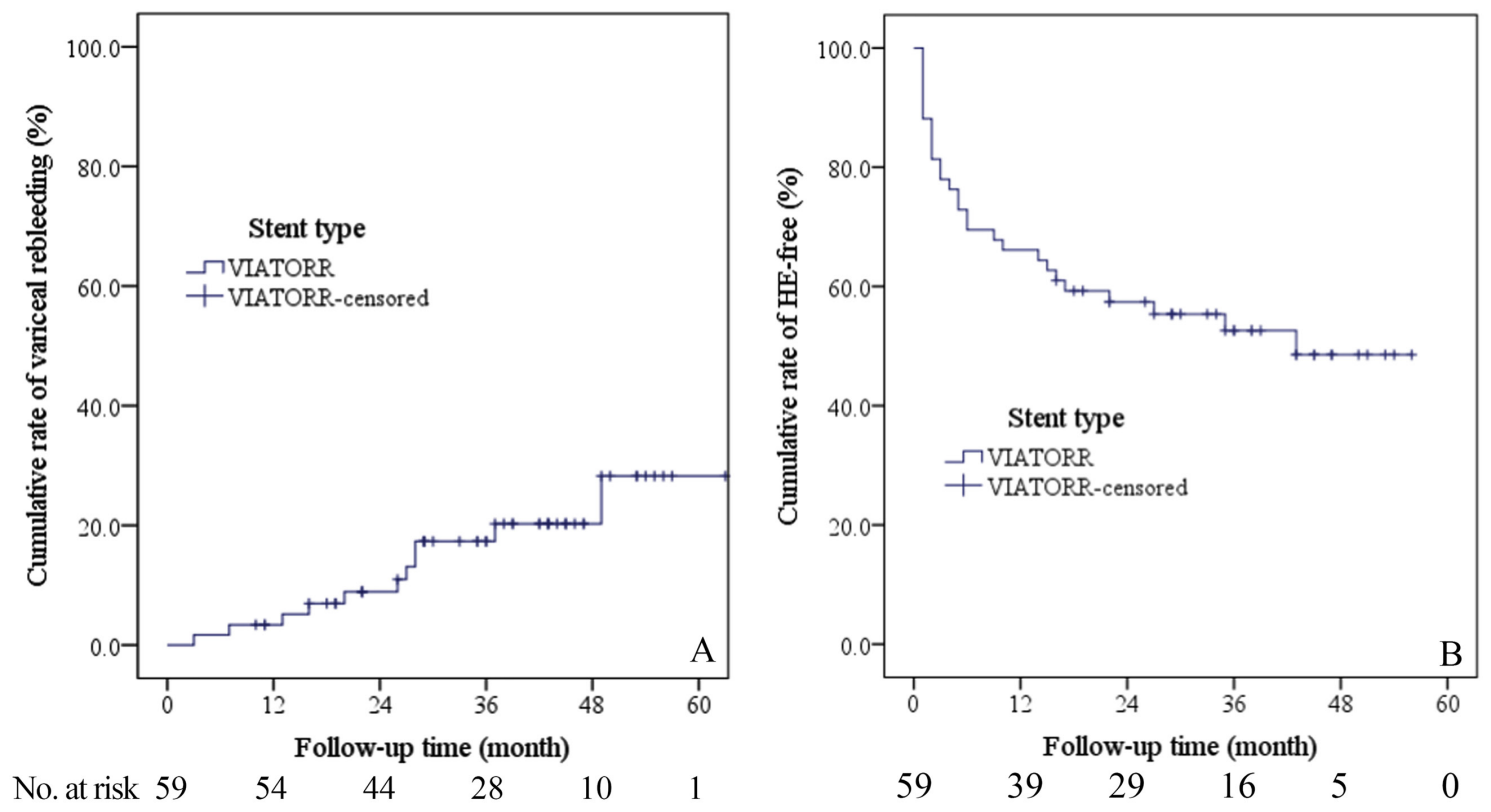
Cumulative rates of variceal rebleeding **(A)**, HE-free patients **(B)**. HE, hepatic encephalopathy.

Overall, eleven patients (18.6%) had variceal rebleeding as confirmed by endoscopy. Six out of the eleven patients with variceal rebleeding were confirmed to have TIPS dysfunction (two with TIPS occlusion and four with shunt stenosis). After successful revision, these six patients did not experience variceal rebleeding until the end of this study. As for the other five patients, four had abnormal ultrasonography (two with slowed blood flow and two with undetectable blood flow within the TIPS) but normal CTPV were subjected to a wait-and-see strategy rather than performing direct portal venography, while the remaining one patient with both abnormal ultrasonography and abnormal CTPV refused to undertake direct portal venography. As a result, the cumulative variceal rebleeding rate at 1, 2, 3, 4 and 5 years was 3.4%, 8.9%, 17.3%, 20.3% and 28.3%, respectively (Figure 3A). Univariate analysis showed that alanine transaminase (ALT), etiology and stent connection were significant factors associated with variceal rebleeding. However, none was still significantly associated with variceal rebleeding in multivariate analysis (Supplementary Table 2).

### Post-Tips Hepatic Encephalopathy

During the study, 28 patients (47.5%) experienced at least one episode of HE, all of which were successfully reversed by conservative treatment with oral lactulose and L-ornithine L-aspartate, and intravenous antibiotics. None needed to be managed by interventional techniques (for example TIPS reduction or occlusion). Therefore, the cumulative 1, 2, 3 and 4-year HE-free rate was 66.1%, 57.4%, 52.6% and 48.6%, respectively (Figure 3B). Univariate analysis revealed that age, comorbidity with type 2 diabetes mellitus (T2DM), albumin, cholinesterase, CTP score and Child class were prominently associated with development of HE. However, in multivariate analysis, only age (HR = 1.048, 95% CI: 0.995–1.103, P = 0.076) tended to be an independent predictor for HE (Supplementary Table 3).

### Safety Profile

Following TIPS implantation, periprocedural complications were observed in eighteen cases: nausea and vomiting (n = 12), venous bleeding from the puncture site at neck (n = 2), hepatic capsular puncture leading to intra-abdominal hemorrhage (n = 2), transient respiratory distress and tachycardia (n = 1). Notably, one male patient developed heamatemesis and melena with obvious decline in hemoglobin and haemotocrit one day after TIPS implantation. He was diagnosed with biliary tract bleeding caused by biliary tract puncture during TIPS procedure, and was successfully treated by ultra-selective hepatic arterial embolization. And he eventually recovered without long-term sequelae.

## Discussion

Recurrent variceal bleeding is the most frequent life-threatening complication of PH. For cirrhotic patients with a prior history of variceal bleeding, the incidence of variceal rebleeding is about 60% within one or two years, and the mortality risk from each rebleeding episode is about 20% [21]. As for secondary prophylaxis of recurrent variceal bleeding, current guidelines recommend pharmacologic treatment with non-selective β-blockers (NSBB), and endoscopic therapy with sclerotherapy and/or variceal band ligation (VBL) as the first-line treatment, and TIPS as a second-line rescue therapy when first-line treatments failed [22,23]. Compared to endoscopic therapy, numerous studies and meta-analysis showed that TIPS was currently a better choice to prevent variceal rebleeding except that TIPS was worse for development of HE [24,25,26].

For patients with variceal bleeding, the primary outcome is to reduce overall mortality. As shown in Table 3, the cumulative mortality rates at 1, 2, 3, 4 and 5 years were 3.4%, 12.3%, 28.9%, 34.2% and 34.2% in our study, which were similar to those reported from China [27], and were slightly lower than reported rates from western countries [24,28]. The discrepant mortality rates may be accountable by different patient selection between Chinese studies and western ones, whereby the major etiology of liver disease and indication of TIPS placement was viral hepatitis and variceal bleeding, respectively, compared to alcoholic liver cirrhosis and a substantial proportion of patients with RA in western studies [24,28]. In our study, direct bilirubin and post-TIPS right atrial pressure were shown to be significant risk factors for mortality, which was in line with previous studies that identified bilirubin level as predictor of survival in TIPS-treated patients with RA [29]. And previous studies demonstrated that TIPS implantation may aggravate the hyperdynamic circulation state due to shunting of blood from the splanchnic vascular bed into the central vascular bed, increasing the risk of heart decompensation and incidence of acute heart failure [30], and higher pre-TIPS right atrial and portal vein pressures were likely to predispose patients to this complication [31], which may explain our finding of post-TIPS right atrial pressure (a known index of cardiac volume) as a risk factor for mortality.

**Table 3:** The clinical outcomes of studies from Western countries and China.


OUTCOMES STUDY	HOLSTER [24]	KRAGLUND [28]	LIN [9]	ZHOU [27]	OUR STUDY

Mortality					

at 1 year	22.7%	18%	—	8.1%	3.4%

at 2 years	22.7%	—	—	8.1%	12.3%

at 3 years	32%	40%	—	—	28.9%

Variceal rebleeding					

at 1 year	0%	23%	0%	0%	3.4%

at 2 years	0%	—	—	9%	8.9%

at 3 years	0%	27%	—	—	17.3%

Shunt dysfunction					

at 1 year	6%	15%	0.95%	5.6%	6.9%

at 2 years	6%	—	—	23.7%	6.9%

at 3 years	—	20%	—	—	11.0%

Hepatic encephalopathy					

at 1 year	35%	38%	38.1%	21.9%	33.9%

at 2 years	38%	—	—	21.9%	42.6%

at 3 years	38%	—	—	—	47.4%


As for variceal rebleeding, our study showed that the cumulative rate was 3.4% at 1 year, 8.9% at 2 years and 17.3% at 3 years (Table 3), which was comparable to the reported rates ranged from 0% to 23% at 1 year and 0% to 27% at 3 years [24,28] in western studies, and 0% at 1 year and 9% at 2 years in Chinese studies [9,27]. This may be explained by the fact that portal pressure gradient is the dominant factor determine variceal bleeding [32], and TIPS can effectively reduce portal hypertension irrespective of patient selection. Our study demonstrated that baseline ALT, etiology and stent connection were significantly associated with variceal rebleeding at univarivate analysis, while none was at multivariate analysis, probably due to its small sample size that was insufficient to identify any predictor.

The efficacy of TIPS implantation is closely related to shunt patency. Table 3 showed that shunt dysfunction rates ranged from 6% to 20% within three years in western countries [24,28], and from 1% to 23.7% within two years in China [9,27]. In line with these studies [9,24,27,28], the shunt dysfunction rate was 6.9%, 6.9%, 11.0%, 11.0% and 11.0% at 1, 2, 3, 4 and 5 years in our study, respectively, suggesting that Viatorr TIPS stent-grafts had similar shunt patency in Chinese patients to western ones.

The major shortcoming of TIPS is development of HE that is associated with increased death risk [33], decreased quality of life of patients and their families [34], and expensive hospitalizations. Previous studies reported that after TIPS implantation (Table 3), HE occurred in 35% to 38% of patients within three years in western countries [24,28], and 21.9% and 38.1% within two years in Chinese studies [9,27]. In agreement with these studies [9,24,27,28], our study demonstrated that the cumulative rate of HE development at 1, 2, 3 and 4 years was 33.9%, 42.6%, 47.4% and 51.4%, respectively. Moreover, Holster IL et al. [24] showed that both male gender (P = 0.004) and TIPS placement (P = 0.033) were independent predictors for HE. Consistently, our study showed that age (HR = 1.048, 95% CI: 0.995–1.103, P = 0.076) tended to be an independent predictor for HE (Supplementary Table 3), probably due to its small sample size.

Our study had several limitations. One major limitation is its single-arm, retrospective design, and thus no direct comparative data can be attained. Another limitation is its small sample size and single centre nature. However, the follow-up period of this study is relatively long. Moreover, there are very limited number of studies about the use of Viatorr TIPS stent-grafts from China, our study definitely adds some referring value to the literature.

In conclusion, elective TIPS implantation (>72 h after variceal bleeding) using Viatorr TIPS stent-grafts is generally safe, and the long-term efficacy is comparable for the treatment of cirrhotic patients with recurrent variceal bleeding or RA in China and western countries.

## Additional File

The additional file for this article can be found as follows:

10.5334/jbsr.2741.s1 Supplementary Tables. Tables 1 to 3.

## Abbreviations

TIPS, transjugular intrahepatic portosystemic shunt; PTFE, polytetrafluoroethylene; HR, hazard ratio; CI, confidence interval; HE, hepatic encephalopathy; VTS, legacy Viatorr TIPS stent-grafts; CTP, Child–Turcotte–Pugh; MELD, model for end-stage liver disease; PT, prothrombin time; INR, international normalized ratio; ALT, alanine transaminase.
