# Supplementary material for: Elective Transjugular Intrahepatic Portosystemic Shunt Using Viatorr Stent-Grafts: A Single-Center Experience from China
Source: J Belg Soc Radiol. 2022 Jun 29;106(1):62. doi: 10.5334/jbsr.2741 (PMC9248993; doi:10.5334/jbsr.2741)
Supplement: Supplementary Tables. — Tables 1 to 3. [file jbsr-106-1-2741-s1.pdf]

Supplementary Table 1 Univariate and multivariate analyses of predictors for shunt dysfunction

Baseline variables	Univariable analysis			Multivariable analysis		
	HR	95%CI	P value	HR	95%CI	P value
Portal venous pressure gradient (mmHg)	1.515	1.221-1.880	0.000	2.572	1.094-6.047	0.030
Stent connection(1)*	0.014	0.001-0.158	0.001			
Etiology(1)#	0.046	0.002-0.868	0.040			
Etiology(2)#	0.012	0.001-0.162	0.001			
Etiology(3)#	0.019	0.001-0.355	0.008			

HR, Hazard ratio; CI, confidence interval. Stent connection(1)*: from middle hepatic vein to left portal branch *vs.* from IVC to portal vein; Etiology#: 1=cryptogenic *vs.* Budd-chiari syndrome, 2=Hepatitis B virus *vs.* Budd-chiari syndrome, 3=autoimmune liver disease *vs.* Budd-chiari syndrome

Supplementary Table 2 Univariate and multivariate analyses of predictors for variceal rebleeding

Baseline variables	Univariable analysis			Multivariable analysis		
	HR	95%CI	P value	HR	95%CI	P value
Alanine transaminase	0.901	0.828-0.981	0.016	0.922	0.850-1.000	0.050

Etiology*	1.885	1.120-3.172	0.017
Stent connection#	1.790	1.031-3.108	0.039

HR, Hazard ratio; CI, confidence interval; etiology*: 0=cryptogenic, 1=alcoholic, 2=hepatitis B virus, 3=hepatitis C virus, 4=autoimmune liver disease, 5=Budd-Chiari syndrome; Stent connection#: 1=from middle hepatic vein to left portal branch, 2= from middle hepatic vein to right portal branch, 3= from right hepatic vein to right portal branch, 4=from inferior vena cava to portal vein.

Supplementary Table 3 Univariate and multivariate analyses of predictors for hepatic encephalopathy

Baseline variables	Univariable analysis			Multivariable analysis		
	HR	95%CI	P value	HR	95%CI	P value
Age	1.054	1.009-1.101	0.019	1.048	0.995-1.103	0.076
T2DM*	0.311	0.143-0.677	0.003			
Albumin	0.902	0.834-0.976	0.010			
Cholinesterase	0.980	0.990-1.000	0.045			
Child score	1.325	1.047-1.678	0.019			
Child class#	1.860	1.083-3.195	0.025			

HR, Hazard ratio; CI, confidence interval; T2DM, type 2 diabetes mellitus；T2DM*：non-T2DM *vs.* T2DM; Child class#: 1=A, 2=B, 3=C.
